# Changes in Topological Organization of Functional PET Brain Network with Normal Aging

**DOI:** 10.1371/journal.pone.0088690

**Published:** 2014-02-20

**Authors:** Zhiliang Liu, Lining Ke, Huafeng Liu, Wenhua Huang, Zhenghui Hu

**Affiliations:** 1 State Key Laboratory of Modern Optical Instrumentation, Department of Optical Engineering, Zhejiang University, Hangzhou, China; 2 Institute of Clinical Anatomy, School of Basic Medical Sciences, Southern Medical University, Guangzhou, China; The University of Chicago, United States of America

## Abstract

Recent studies about brain network have suggested that normal aging is associated with alterations in coordinated patterns of the large-scale brain functional and structural systems. However, age-related changes in functional networks constructed *via* positron emission tomography (PET) data are still barely understood. Here, we constructed functional brain networks composed of 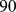 regions in younger (mean age 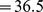 years) and older (mean age 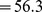 years) age groups with PET data. 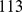 younger and 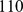 older healthy individuals were separately selected for two age groups, from a physical examination database. Corresponding brain functional networks of the two groups were constructed by thresholding average cerebral glucose metabolism correlation matrices of 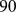 regions and analysed using graph theoretical approaches. Although both groups showed normal small-world architecture in the PET networks, increased clustering and decreased efficiency were found in older subjects, implying a degeneration process that brain system shifts from a small-world network to regular one along with normal aging. Moreover, normal senescence was related to changed nodal centralities predominantly in association and paralimbic cortex regions, *e.g.* increasing in orbitofrontal cortex (middle) and decreasing in left hippocampus. Additionally, the older networks were about equally as robust to random failures as younger counterpart, but more vulnerable against targeted attacks. Finally, methods in the construction of the PET networks revealed reasonable robustness. Our findings enhanced the understanding about the topological principles of PET networks and changes related to normal aging.

## Introduction

Function decline and organs aging is an inevitable physiological law of life. As one of the most important organs, aging brain tends to produce some specific alterations in morphological, physiological pathology and functional aspects. It is well known that normal aging is associated with a progressive decline in cognitive performance, including perception, attention, language and memory [1], [2], [3], [4]. Meanwhile, normal senescence is also highly related to some specific encephalopathies, such as Alzheimer’s disease (AD) [5] and Parkinson’s disease (PD) [6]. Therefore, aiming to assess the declining cognitive ability and supply a guiding for age-related encephalopathy in clinical, it is necessary to deeply understand the age-related changes in healthy brain.

An emerging approach for studying human brain system is graph theory which is represented by a set of nodes and links. It has been widely adopted to quantify complex system, *e.g.*, in social sciences, biology or technology [7], [8]. The functional and structural systems of the human brain reveal age-related topological properties of complex networks, such as small-world characteristics, highly connected hubs, modularity, and network robustness [7], [9], [10], [8], [11]. Small-world properties, characterized by a high degree of clustering and a short average distance between any two nodes [12], [13], were analysed to reveal age-related global and local efficiency of information transfer in brain system. Some recent studies reported that, along with the normal aging, small-world network showed changed topological efficiency [10], [14], [15], [16]. For instance, a recent study on functional brain networks suggested that an older age group showed significantly reduced cost efficiency in comparison to a younger group [17]. A similar degeneration process of economical small-world networks was also found in a previous study about AD [18], [19]. Furthermore, changes in modular organization of human brain networks were proven to be associated with normal aging [9], [15]. Simultaneously, highly connected hubs are altered with normal aging, which has been reported in some previous studies [9], [7]. In addition, previous studies also found that normal aging processes significantly affect default mode network (DMN) [20], [21], [22], [23], which is typically deactivated during external stimulation [24], [25]. Thus, a gradually forming evaluated system of brain networks with neural imaging technologies was adopted in assessing the aging brain and provided a guiding for age-related encephalopathy in clinical [7], [8].

However, up to now, few studies have constructed functional network *via* positron emission tomography (PET) data. Compared with other functional signals, PET can offer a more immediate way to indicate brain activity by offering the index of cerebral glucose metabolism. In this paper, for evaluating the age-related brain changes in normal individuals, the large-scale human brain functional network was constructed by node, defined as regional average cerebral glucose metabolism from PET data. Four main reasons indicate that the definition is reasonable and effective for assessing age-related brain functional changes. Firstly, during the resting state, the level of cerebral glucose metabolism is considered as a reliable index of neural activity [48]. Secondly, synapses are considered as the key sites for transferring information between neurons, and up to 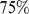 of the glucose consumption in the brain is used to maintain a baseline synaptic activity [27]. Thirdly, many studies have reported that the normal aging is accomplished by a decline of synaptic activity, which impacts the cognitive functions [28], [29]. Lastly, effective connectivity between PET regions has been found in previous studies [30], [31]. Thus, the definition of PET nodes can provide a complementary and convincible way to improve the evaluation of brain functional networks.

In the present study, large-scale functional networks (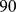 regions) in two age groups (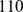 older subjects, 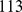 younger subjects) were constructed by computing the partial correlation matrices of the regional mean intensity values from PET data. Afterwards, we investigated the brain functional topological properties, including small-world characteristics, hub regions and network robustness, revealing the brain functional changes associated with normal aging. Methodological robustness in the construction of PET network was also assessed.

## Materials and Methods

### Subjects

Two hundred and twenty-three healthy human subjects were selected from a physical examination database, and written informed consents for the future research were obtained from all subjects. They were separated into two age groups, 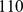 older subjects aged 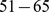 years (mean age = 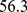 years, 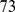 male) and 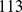 younger subjects aged 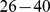 years (mean age = 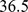 years, 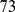 male). Health status of all subjects were evaluated with a normal physical examination before imaging. Individuals with significant chronic or acute disease were excluded from subjects. Other criteria of subjects’ physical condition were as follows: native Mandarin Chinese speaker, right-handed, same average education years, no history of neurological disease, no brain trauma or clinical evidence of cognitive impairment.

### PET Data Acquisition

All PET investigations were implemented with Hamamatsu SHR 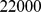 whole-body PET scanner system located at the Medical PET Center of Zhejiang University. The spatial resolution of the scanner is 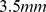 full width at half maximum (FWHM) in sagittal or coronal plane and 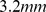 FWHM in axial plane. There is a 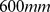 patient aperture and an axial field-of-view of 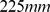 in the scanner, which can deal with the whole head. A 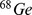 source for attenuation correction was used in the emission scan after a 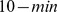 transmission scanning. All subjects were injected intravenously with 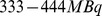 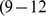 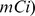 fluorodeoxyglucose (FDG) before resting in a dark, quiet room with ears open and eye closed for 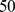 minutes. Then each subject was scanned lying quietly at rest with eyes closed for 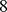 minutes. Nyquist frequency was acquired, after a ramp filter with the maximum-likelihood expectation maximization (MLEM) algorithm was used in reconstructions of PET images. Additionally, the acquisitions were performed with the approval of the Health Science Research Ethics Committee of Zhejiang University.

### PET Data Preprocessing and Regional Parcellation

After using ImageJ (Wayne Rasband, National Institute of Mental Health, USA) and MRIcro software (http://www.mricro.com), Analyze Formats were acquired from raw PET data. Then the preprocessing was performed using matlab 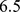 (MathWorks Inc., Notich, MA, USA) and Statistical Parametric Mapping (SPM5, Wellcome Department of Cognitive Neurology, London, UK) software. Each data was normalized into the Montreal Neurological Institute (MNI, McGill University, Montreal, Canada) standard template using SPM5. An isotropic Gaussian kernel with 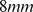 FWHM was used in spatial smoothing to increase the signal-to-noise ratio after normalization. Then the proportional scaling was used for the intensity normalization [32], [33], [34]. Regional parcellation was completed using the anatomically automatic labeled (AAL) template image previously validated by Tzourio-Mazoyer *et al.* [35]. The brain was divided into 90 anatomical regions of interest (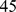 regions for each hemisphere) using this parcellation. Then we calculated mean intensity values of each region which represented the regional cerebral metabolic rates for glucose.

### Partial Correlation Analysis

**Figure 1:**
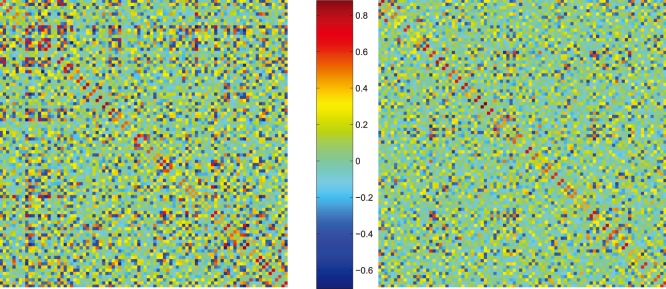
The correlation matrices of two groups. The graphs show the correlation matrices acquired by calculating partial correlations (left for the older group and right for the younger group). The color bar in the middle indicates the partial correlation coefficient between regions. The rank and row successively represent the 90 brain regions (see Table S1).

The functional connection was defined as statistical associations in the intensity values across subjects. Such a connection concept has been introduced by the previous studies [36], [37]. It is reasonable to investigate brain functional systems (networks) by calculating connectivity of the PET regions, since effective connectivity between PET regions has been found previously [30], [31]. So, the analytical procedure was performed by analysing the regional relation obtained above. The interregional correlation matrix 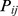 (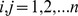, here 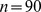) of each group (Figure 1) was acquired by calculating the partial correlation coefficients across individuals between the mean intensity value of every pair of regions. The conditional dependences of arbitrary two regions partialled out the effects of the other 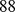 regions defined in the AAL template were represented by the partial correlations between them.

### Construction of Functional Network

**Figure 2:**
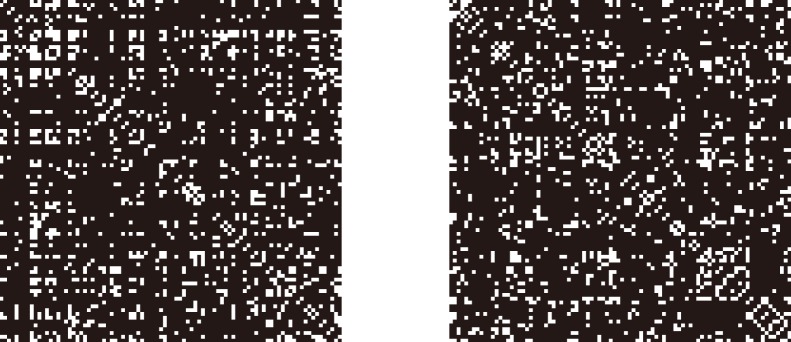
The binarized matrices (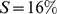) of two groups. The graphs show the binarized matrices (left for the older group and right for the younger group) which are generated by setting threshold to the correlation matrices. The rank and row successively represent the 90 brain regions (see Table S1). Such a threshold (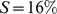) ensures that the networks of both of the groups have the same number of nodes and links, and also show changed efficiency of information transfer (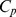, 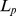, 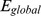). In this graph, white and black indicate the 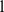 and 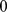.

According to the prior studies [32], [7], functional networks of both groups could be acquired from their partial correlation matrices. The partial correlation matrix of each group was converted into a binarized matrix 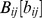 (Figure 2) by setting a threshold. Element of 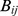 was 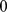 if the absolute value of the correlation between regions 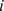 and 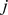 was smaller than given correlation threshold and 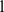 otherwise. Topological organization of the human functional networks was represented by the binary matrices. Then a binary graph theoretical analysis [7], [8] was performed in the following.

### Graph Theoretical Analysis

#### Sparsity selection

To perform a graph theoretical analysis, the binarized matrix 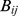 is described as a network (graph) 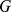 defined by 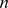 nodes and 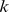 edges, where nodes indicate regions and edges indicate undirected links between regions according to nonzero elements of 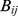. A fixed sparsity 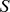 of each network, which was defined as the total number of edges 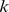 in a graph divided by the maximum possible number of edges, would be calculated after its correlation matrix was thresholded into the binarized matrix. It was stated briefly that same correlation threshold would lead to different number of edges (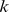, or 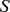) between the both resulting graphs because of the difference in the low-level correlations (see Figure 1). Therefore, alterations in the topological organization would not be solely reflected by the between-group difference in network parameters. Hence, a sparsity-specific threshold was set to ensure that the both undirected graphs had the same number of edges (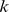) or wiring cost [17], [18]. Because a single and definitive threshold could not be selected currently, graphs with wide range of sparisty (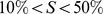) was generated by repeatedly thresholding each correlation matrix, then properties of them were estimated at each threshold value. Then small-world parameters between the two groups were compared as a function of independent sparsity of the precise selection of threshold. The range of sparisty (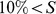) also ensured that every nodal pairs in both graphs had a connecting path (mentioned below) [38]. Then we estimated network properties including clustering coefficient, path length, global efficiency, nodal centrality and network robustness in the following steps.

#### Clustering coefficient

**Figure 3:**
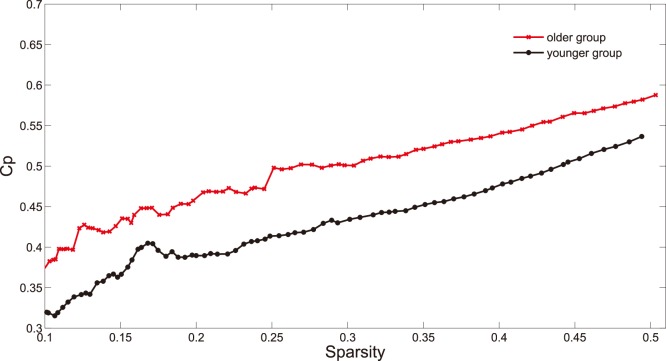
Clustering coefficient (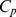) as a function of sparsity. The graph shows that, at a wide range of sparsity (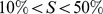), the older subjects (red line) have larger 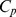 value than the younger subjects (black line).

A cluster of node 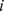 is formed by directly connected nearest neighbours of the node [7]. Clustering coefficient 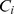 of a node 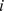 quantifies the number of connections existing in the cluster as a proportion of maximum possible connections [12]. 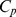 (Figure 3) of a network is defined as the average of 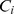 over all nodes in a network and indicates the extent of local cliquishness or local efficiency of information transfer [12], [13].

#### Path length and global efficiency

**Figure 4:**
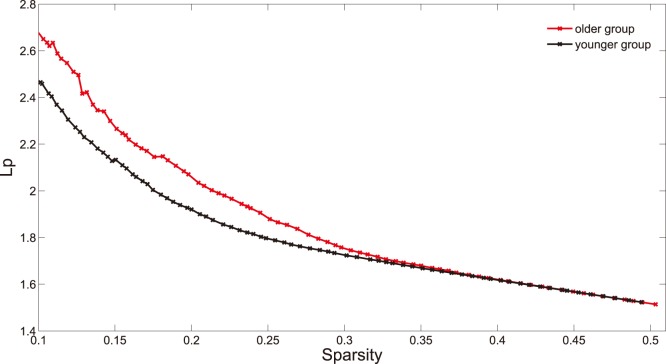
Path length (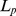) as a function of sparsity. The graph shows that two groups have same 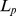 value when sparsity ranges from 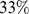 to 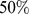 and the older group (red line) have larger 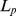 at 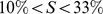.

**Figure 5:**
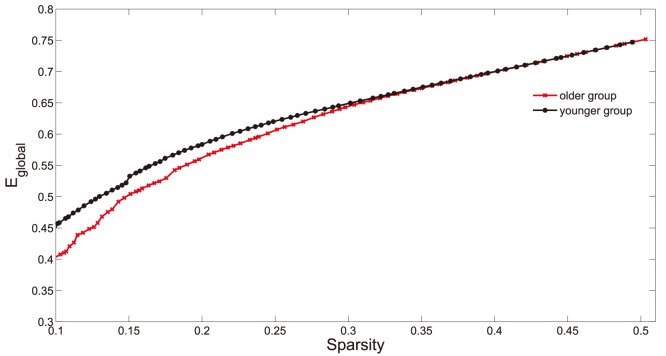
Global efficiency (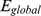) as a function of sparsity. 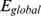
 is numerically easier to indicate the global efficiency than 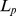 (see Material and Methods). As the sparsity thresholds increase from 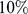 to 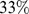, 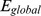 of both groups increase, and younger subjects (black line) have larger 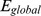 values. At high sparsity threshold (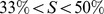), two groups show equal 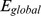 values.

Path length 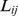 between node 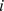 and node 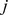 is defined as the minimum number of edges traversed from node 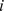 to node 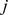. 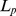 (Figure 4) is defined as the average 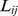 of the all pairs nodes of the network and quantifies the ability of global efficiency of parallel information transfer [13]. Global efficiency (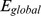, Figure 5) inversely related to 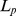 but numerically easier to indicate the global efficiency of parallel information transfer was also estimated. Global efficiency (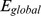) measure is 
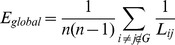
, 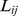 means the path length of the node 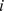 and node 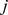. Of note, the problem about the definition of 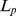 caused by nodal pairs without connecting path, can been eliminated by the range of sparisty (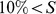, mentioned above).

#### Small-world analysis

**Figure 6:**
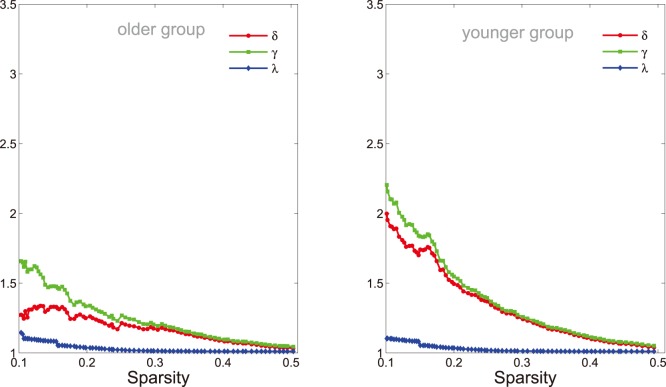
Small-world parameters of networks. The graphs show the changes in 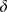 (red line), 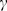 (green line) and 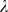 (blue line) in the networks of older (left panel) and younger (right panel) groups as a function of sparisty thresholds. At a wide range of sparsity, both networks have 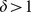, that implies prominent small-world properties (see Materials and Methods). Note that, as the values of sparsity thresholds increase, the 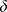 and 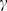 values decrease rapidly, but the 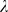 values decrease rapidly when sparsity range from 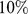 to 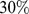 then change slightly.

In this study, small-world properties of networks in two groups were examined according to the 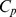 and 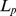 measured in the above steps. A small-world network should meet the following criteria: 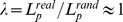 and 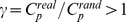 [12], or 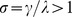 [39], [40](see Figure 6), where the 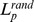 and 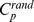 are the mean path length and clustering coefficient of 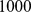 suitable random networks with the same number of nodes, edges, and degree (the degree 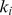 of a node 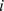 is the number of connections to that node) as the real network [12], [41]. Random graphs were generated by the random rewiring procedure [42], [43].

#### Betweeness centrality

**Figure 7:**
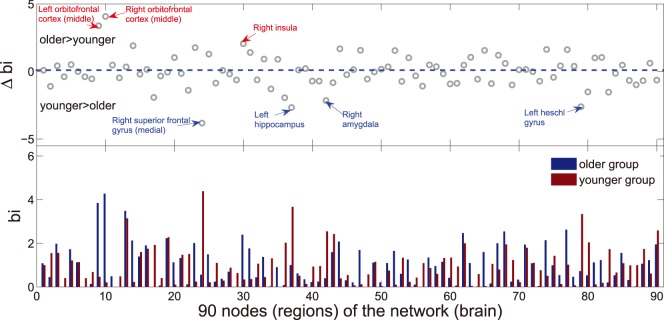
Betweenness centrality (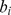) of two groups. The below graph shows the comparison (red bar for younger group and blue bar for older group) of normalized betweenness (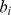) in each node (region) between two groups. The upper graph shows the regional changes (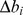, 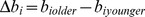) in normalized betweenness (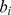) between two groups. The regions labeled in the upper graph indicate significant changes in 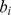 between two groups (see Table 4). Note that these results were obtained from the brain networks with a sparsity of 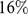. Regions in networks of two groups showing high 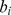 value (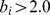) have been listed in the Table 2 and Table 3.

The centrality (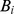) of node 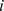 is defined as the number of the shortest paths between all other node pairs pass through it [44]. A node with high value 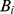 is crucial to efficient communication in the network and is considered as the hub of the network [7]. Here 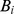 was calculated by using the MatlabBGL package (http://www.stanford.edu/∼dgleich/programs/matlab_bgl/). Then normalized betweenness (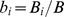, see Figure 7) was measured to estimate nodal characteristics of the networks, where 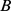 was the average 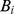 over all nodes in the network.

#### Network robustness

**Figure 8:**
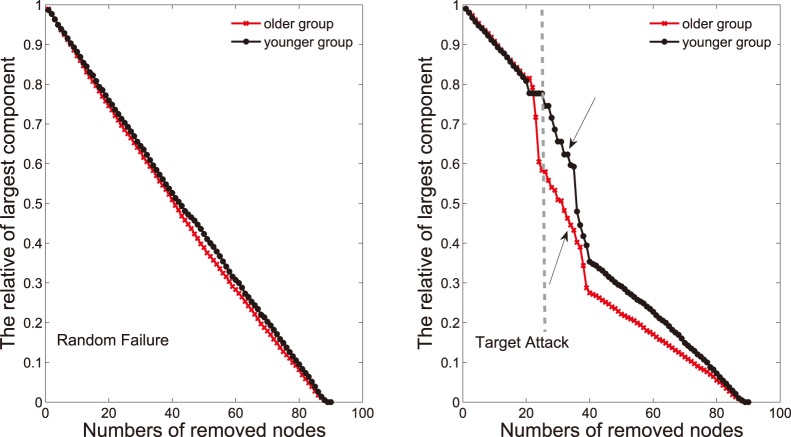
Topological robustness in networks of two groups. The graphs show the relative size of the largest connected component as a function of the fraction of removed nodes by random failures or targeted attacks. As the response to random failures (left panel), the brain network in the older group (red line) is approximately as robust as that in the younger group (black line). Right graph shows that the older network displays remarkably reduced stability against targeted attack compared with the younger. Additionally, the statistical significant differences (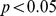) of two groups was found with the ranges of 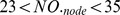 and 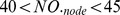 in the right graph.

In this step, a simple analysis about network robustness was performed. Network robustness associated with the stability of a complex network refers to the degree of tolerance against random failures and targeted attacks [7]. In the current studies, robustness (tolerance) of the networks was investigated through removing nodes in the networks [45], [46], [40]. Firstly, to test the the nodal failure tolerance, one node was removed from the networks and changes in the size of the largest connected component were measured. Then other nodes were removed sequentially at random (Figure 8). To address the attack tolerance, the above processes were repeated but we removed the nodes of high 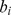 value in the targeted position (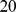 nodes of high 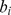 value were removed from 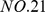 to 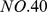 in abscissa axis, showed in Figure 8). To investigate the comparison of the network robustness between two groups, the procedures were repeated 1000 times for the networks of both groups. Then we calculated the mean relative of largest component (Figure 8). Additionally, in order to investigate statistical differences, the 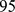 percentile points of each distribution were used as the critical values for a two-tailed test of the null hypothesis with a probability of type I error of 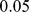, with every number of the removed nodes under two attacks.

### Statistical Analysis

#### Correlation differences in statistical analysis

It is necessary to validate the significance difference of these correlations in two groups with statistical analysis of correlations between 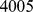 pairs of regions. The 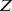 values approximately normally distributed were generated from correlation coefficients, after Fishers 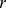-to-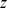 transform. Then the transformed 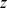 values were compared by 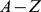 statistic to determine the significance of the between-group differences in correlations [47]. A false discovery rate (FDR) procedure [48] was performed to adjust to the the multiple comparisons at a 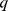 value of 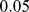.

#### Statistical differences in topological parameters

A nonparametric permutation test method was applied to determine statistical significance of the between-group differences. First of all, 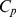, 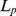, 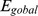 and 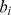 of the two-groups networks with a given sparsity were separately computed. Secondly, to test the null hypothesis that the group differences might occur by chance, we then randomly reallocated each individual set of regional cerebral glucose metabolism to one or the other of the two groups. Thirdly, after recomputing the correlation matrix and obtaining binarized matrix, we recalculated the network parameters for each randomized group, using the same method. Lastly, this randomization procedure was repeated 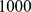 times and the 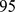 percentile points of each distribution were used as the critical values for a one-tailed test of the null hypothesis with a probability of type I error of 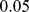. Then the procedure was repeated at every sparsity threshold value of the networks.

### Methodological Robustness Analysis

**Figure 9:**
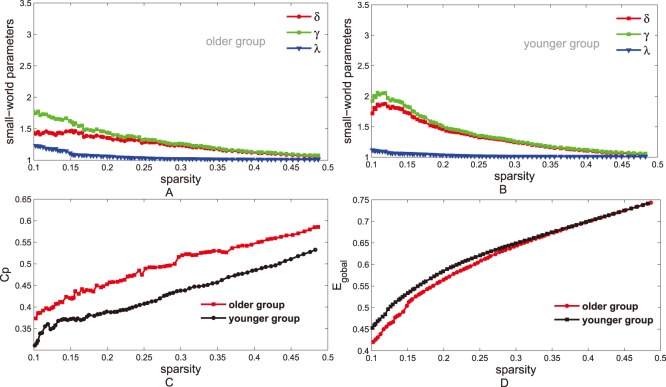
Small-world properties in smaller-sample networks. **A** and **B**, The graphs show the small-world parameters (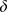, 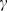 and 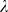) of smaller-sample networks in older (**A**) and younger (**B**) groups. At a wide range of sparsity, both networks have 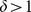, that implies prominent small-world properties (see Materials and Methods). **C,** This graph shows that older subjects (red line) have larger 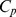 values than the younger subjects (black line). In the original networks, similar result of 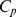 was also shown in Figure 3. **D**, This graph shows the global efficiency (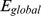) as a function of sparsity. As the sparsity thresholds increase from 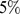 to 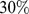, 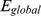 of both groups increase and younger subjects (black line) have larger 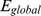 values.

It is necessary to test the methodological robustness in the construction of networks, because the PET networks are barely constructed by calculating the partial correlation matrices. In this study, the methodological robustness was estimated by reducing sample size in each group. Firstly, 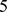 individuals were separately removed from the both groups at random, to test methodological robustness against the reductions of samples size in both groups. It is noted that quantities (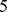 nodes) of the removal individuals are limited by the size of the whole sample. After the above step was repeated 50 times, the mean smaller-sample networks (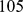 in older group and 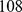 in younger group) were obtained. As a comparison to the former networks, the small-world parameters (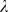,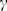 and 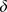, see Figure 9 **A** and **B**), global efficiency (Figure 9 **D**) and local efficiency (Figure 9 **C**) in smaller-sample networks were calculated via the above methods. Furthermore, in order to determine statistical significance of the neo-networks differences, the nonparametric permutation test method was applied on 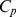 and 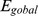. Methodological robustness was analysed by comparing the small-world properties between networks with different samples.

## Results

### Correlations of Regions in Two Groups

As is shown in Figure 1, the interregional correlation matrices represent complex correlation patterns of both age groups. Statistical analysis further demonstrates significant between-group correlation differences (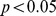, FDR-corrected) in various pairs of regions (Table 1). For instance, older subjects are found to show increased positive correlations in various pairs of cortical regions involved in the frontal, precentral and postcentral. In addition, decreased positive correlations in older group are observed in pairs of regions including hippocampus, amygdala. We also note several changed negative correlations in the older group. All pairs of regions with significant between-group changed correlation coefficients are listed in Table 1.

**Table 1:** Comparisons of correlation between two groups.

Regions		Correlation, 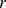 , ( 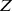 )	
		Young	Old
**Increased positive correlation in older group**			
Left orbitofrontal cortex (middle)	Left supplementary motor area	−0.15(−0.15)	**0.66(0.79)**
Left orbitofrontal cortex (superior)	Right inferior parietal lobule	−0.08(−0.08)	**0.72(0.91)**
Left orbitofrontal cortex (middle)	Right orbitofrontal cortex (inferior)	0.03(0.03)	**0.57(0.65)**
Left orbitofrontal cortex (middle)	Left orbitofrontal cortex (superior)	0.04(0.04)	**0.56(0.63)**
Left orbitofrontal cortex (middle)	Left olfactory	0.03(0.03)	**0.57(0.65)**
Left precental gyrus	Left supplementary motor area	0.02(0.02)	**0.62(0.73)**
Right precental gyrus	Right supplementary motor area	0.13(0.13)	**0.65(0.78)**
Left inferior frontal gyrus (opercular)	Right supplementary motor area	0.01(0.01)	**0.53(0.59)**
Right orbitofrontal cortex (middle)	Left superior frontal gyrus (medial)	0.12(0.12)	**0.64(0.76)**
Left olfactory	Left superior frontal gyrus (medial)	0.04(0.04)	**0.60(0.69)**
Left inferior frontal gyrus (triangular)	Right inferior parietal lobule	0.09(0.09)	**0.64(0.76)**
left postcentral gyrus	Left supramarginal gyrus	0.10(0.10)	**0.61(0.71)**
**Decreased positive correlation in older group**			
Left hippocampus	Left amygdala	**0.72(0.91)**	−0.13(−0.13)
Left superior frontal gyrus (dorsal)	Left middle frontal gyrus	**0.62(0.73)**	−0.23(−0.23)
Right rolandic operculum	Right precuneus	**0.58(0.66)**	0.06(0.06)
**Increased negative correlation in older group**			
Right orbitofrontal cortex (inferior)	Left supplementary motor area	0.01(0.01)	−**0.65(**−**0.78)**
Right inferior frontal gyrus (triangular)	Left superior frontal gyrus (medial)	0.11(0.11)	−**0.64(**−**0.76)**
Left orbitofrontal cortex (middle)	Left posterior cingulate gyrus	−0.03(−0.03)	−**0.62(**−**0.73)**
Left orbitofrontal cortex (middle)	Right inferior parietal lobule	−0.14(−0.14)	−**0.66(**−**0.79)**
Left orbitofrontal cortex (middle)	Left middle occipital gyrus	−0.01(−0.01)	−**0.53(**−**0.59)**
Left orbitofrontal cortex (middle)	Right putamen	−0.04(−0.04)	−**0.57(**−**0.65)**
Right middle occipital gyrus	Right precuneus	−0.08(−0.08)	−**0.59(**−**0.68)**
Left thalamus	Right orbitofrontal cortex (middle)	−0.08(−0.08)	−**0.61(**−**0.71)**
Left thalamus	Left olfactory	−0.03(−0.03)	−**0.60(**−**0.69)**
Left thalamus	Right calcarine cortex	−0.01(−0.01)	−**0.58(**−**0.66)**
Right paracentral lobule	Right superior temporal gyrus	0.01(0.01)	−**0.56(**−**0.63)**
Right hippocampus	Left temporal pole (superior)	0.04(0.04)	−**0.61(**−**0.71)**
Left caudate	Right middle temporal gyrus	0.03(0.03)	−**0.56(**−**0.63)**
Right precuneus	Right Temporal pole (middle)	0.01(0.01)	−**0.56(**−**0.63)**
**Decreased negative correlation in older group**			
Left rolandic operculum	Right calcarine cortex	−**0.55(**−**0.62)**	−0.01(−0.01)
Left rolandic operculum	Right precuneus	−**0.61(**−**0.71)**	−0.09(−0.09)
Left paracentral lobule	Left heschl gyrus	−**0.54(**−**0.60)**	0.01(0.01)

This table lists pairs of regions with significant changed coefficients (change is larger than 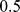) between two groups. Coefficients in bold represent significant interregional association within group. The comparison of coefficients between two groups are also shown in Figure 1. To determine the significance of between-group differences in correlation, a 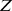 statistic was used in this study (see Materials and Methods). All 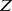 value are significant (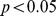, FDR-corrected).

### Small-world Topology Functional Networks

It has been demonstrated in the previous studies [32], [49], [41] that functional network of humans has small-world characteristics. In a small-world network, the nodes of the network have larger local interconnectivity than a random network, but the shortest path length between any pair of nodes is approximately equivalent to a comparable random network [12]. The small-world attributes of the functional networks in two age groups were also examined in the current study. As expected, both functional networks demonstrate small-world characteristics (Figure 6, left for older group and right for younger group, 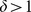 red line) over a wide range of sparsity (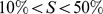). Compared with the matched random networks, they have larger local cliquishness (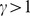, green line) but an almost identical path length (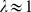, blue line). Using computational modeling simulation approaches, Sporns *et al.* [50] propose the emergence of small-world topology when networks are evolved for high complexity of dynamic behavior defined as an optimal balance between global integration and local specialization. Therefore, our findings additionally support hypothesis that human brain has evolved into a complex but efficient neural architecture to maximize the power of information processing [41], [51].

### Different Small-world Parameters between Two Age Groups

As shown in Figure 3, clustering coefficient (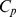) in networks of older group (red line in Figure 3) are larger than those of younger group (black line in Figure 3) over a wide range of sparsity (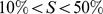). Global efficiency (Figure 5) in networks of younger group (red line in Figure 5) are larger than those of older group (black line in Figure 5), when sparsity ranged from 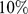 to 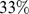. Both groups show different small-world parameters, reduced global efficiency (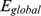, Figure 4 and Figure 5) and increased local cliquishness (Figure 3) in older group. Additional statistical analysis also reveals significant differences (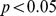) in the 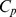 values at 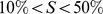, 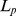 values at 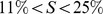 and 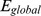 at 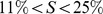. These results imply that older subjects are probably related to the loss of small-world characteristics in the large-scale functional brain systems. In addition, approximate results were obtained in previous studies about normal aging and even AD [32], [16], [18], [19].

### Hub Regions

The functional networks were constructed at a sparsity threshold of 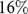 to investigate the nodal characteristics of each region in two age groups. After normalized betweenness centrality (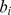) of each region (Figure 7) in both networks was measured (see Materials and Methods), hubs were defined as the regions with high betweenness centrality (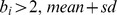). In the older group, 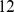 regions (Table 2) are identified as the hubs because of large values in 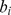. In another group, 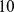 regions are identified as the hubs (Table 3). Our finding of some hub regions (including supplementary motor area, left hippocampus,) is consistent with a previous brain functional network study age-related changes [9].

**Table 2:** Regions showing high betweenness (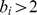) in the network of older group.

Regions	Abbreviations	Class	*b_i_*	Degree, *k_i_*
**Left orbitofrontal cortex (middle)**	ORBmid.L	paralimbic	3.84	41
**Right orbitofrontal cortex (middle)**	ORBmid.R	paralimbic	4.27	37
Left inferior frontal gyrus (triangular)	IFGtriang.L	association	3.48	29
Right inferior frontal gyrus (triangular)	IFGtriang.R	association	2.11	24
Left supplementary motor area	SMA.L	association	2.23	30
Left superior frontal gyrus (medial)	SFGmed.L	association	2.00	23
**Right insula**	INS.R	association	2.39	24
Right calcarine cortex	CAL.R	primary	2.07	27
Right inferior parietal lobule	IPL.R	association	2.46	28
Right precuneus	PCUN.R	association	2.53	33
Right putamen	PUT.R	subcortical	2.13	29
Left thalamus	THA.L	subcortical	2.61	28

This table lists the hub regions (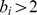) in the network of older group. Regions in bold show increased normalized betweenness (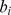) in older group compared with younger group (see Table 4). 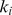 denotes the the degree of region 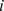. Note that these results were acquired from the brain networks with a sparsity of 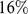.

**Table 3:** Regions showing high betweenness (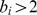) in the network of younger group.

Regions	Abbreviations	Class	*b_i_*	Degree, *k_i_*
Left inferior frontal gyrus (triangular)	IFGtriang.L	association	3.14	22
Left supplementary motor area	SMA.L	association	2.28	18
**Right superior frontal gyrus (medial)**	SFGmed.R	association	4.38	27
Right posterior cingulate gyrus	PCG.R	paralimbic	2.02	12
**Left hippocampus**	HIP.L	paralimbic	3.67	24
**Right amygdala**	AMYG.R	paralimbic	2.54	22
Left calcarine cortex	CAL.L	subcortical	2.42	29
**Left heschl gyrus**	HES.L	association	3.33	32
Right heschl gyrus	HES.R	association	2.03	23
Right inferior temporal gyrus	ITG.R	association	2.58	26

This table lists the hub regions (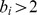) in the network of younger group. Regions in bold show decreased normalized betweenness (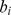) in older group compared with younger group (see Table 4). 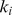 denotes the degree of the region 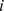. Note that these results were acquired from the brain networks with a sparsity of 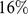.

### Changed Regional Nodal Characteristics between Two Groups

The regions (Figure 7) with between-group changes in betweenness centrality are examined in this study. Compared with the younger subjects, the older show increased betweenness centrality (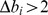) in 3 regions (ORBmid.L, ORBmid.R, INS.R, listed in Table 4) and decreased betweenness centrality in 4 regions (SFGmed.R, HIP.L, AMYG.R, HES.L, listed in Table 4). Additional statistical analysis reveals significant differences (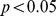) in betweenness centrality of these regions. The changed nodal characteristics (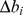) of each region are also showed in Figure 7 (upper panel). Together, our findings suggest that the roles of regions in managing information are profoundly affected by age [9].

**Table 4:** Regions showing significant changes in normalized betweenness (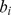) between two groups.

Regions	Abbreviations	Normalized betweenness, *b_i_*		Δ*b_i_*
		Old group	Young group	
**Increased ** 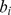 ** in old group**				
Left orbitofrontal cortex (middle)	ORBmid.L	**3.84**	0.47	+3.37
Right orbitofrontal cortex (middle)	ORBmid.R	**4.27**	0.20	+4.07
Right insula	INS.R	**2.39**	0.33	+2.06
**Decreased ** 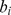 ** in old group**				
Right superior frontal gyrus (medial)	SFGmed.R	0.55	**4.38**	−3.83
Left hippocampus	HIP.L	0.99	**3.67**	−2.68
Right amygdala	AMYG.R	0.39	**2.54**	−2.15
Left heschl gyrus	HES.L	0.72	**3.33**	−2.61

This table shows regions with changes in normalized betweenness (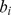) between two groups. Normalized betweenness (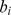) in bold indicate the betweenness centrality of the hub regions which are also showed in Table 2 and Table 3.

### Reduced Network Robustness in Older Subjects

Figure 8 shows the network robustness of two age groups under the targeted attack and random failures. Both groups reveal similar network robustness to the random failures (Figure 8). When the nodes were randomly removed, the sizes of the largest connected component in both groups reduced steadily and approximately (Figure 8, left). Although network robustness of both groups reduced sharply due to the removing of 20 central nodes from 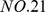 to 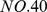, the younger network displayed remarkably stability against targeted attack compared with the older (Figure 8, right). In addition, the statistical significant differences (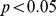) of two groups are only found in the targeted-attack procedure. The specific ranges are 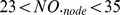 and 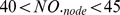.

### Small-world Parameters in Smaller-sample Networks

Figure 9 shows the methodological robustness, in response to the decrease of samples size in both age groups. In the smaller-sample networks, small-world characteristics are also revealed according to 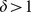 (red lines) shown in Figure 9 **A** and **B**. Simultaneously, larger local efficiency (Figure 9 **C**) and lower global efficiency (Figure 9 **D**) in older group are found in smaller-sample networks. Additional statistical analysis reveals significant differences (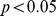) in the 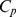 values at all range (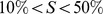), and 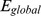 values at 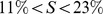. These findings of two groups are compatible with the former results in the original networks.

## Discussion

The current study, for the first time, demonstrates age-related changes in the topological organization of large-scale functional brain networks by utilizing PET data. Our main results are as follows: (1) that the observed data demonstrate age-related alterations in functional correlations among selective subsets of regions, (2) that the global topological organization of functional networks in older subjects are disrupted as indicated by altered small-world parameters, (3) that the regional nodal characteristic (centrality) is changed in older subjects, (4) that the functional network of older group shows reduced network robustness in response to the targeted attack, (5) that the methods to construct the functional PET networks demonstrate reasonable robustness.

### Small-world Characteristics and Age-related Changes

Our findings of high global and local efficiency in functional brain networks with both age groups are consistent with some previous studies [32], [7], [17], [52], [53]. Especially, another PET study [32], which compared the properties of whole-brain functional networks of normal, mild cognitive impairment (MCI) and AD individuals by using FDG-PET data, has reported that brain functional PET networks of all show small-world property. Experimental studies [54] and computational modeling simulations approaches [50] have also proposed the emergence of small-world topology when networks evolved for high complexity of dynamic behavior defined as an optimal balance between global integration and local specialization [55]. Thus, Our findings provide additional support for the hypothesis that the human brain has evolved to maximize the cost efficiency of parallel information processing [50], [51].

We also find age-related changes of global and local efficiency (Figure 3, Figure 4 and Figure 5) in the functional networks. The network may develop into a more local and less distributed organization, in the normal processes of brain senescence. This phenomenon suggests a degeneration process with normal aging, which has been reported in some previous studies [7], [41]. It has been proposed that in comparison to small-world networks, the lattice-like networks have a slow signal propagation speed and synchronizability [55]. Many psychiatric and neurological disorders described as dysconnectivity syndromes are associated with the regular topological organization that disturbs the optimal balance of a small-world network [56]. Previous studies have proposed the regular topological organization of brain networks in patients with diseases such as AD or schizophrenia [52], [18]. These convergent evidences from methodologically disparate studies suggest that both AD and schizophrenia are related to abnormal topological organization of structural and functional brain networks [7]. Therefore, our finding about the degeneration process shows that normal aging has high risk for dysconnectivity syndromes.

In particular, the above results in functional brain networks are conformed to a previous study about structural brain networks [16], which the middle group (mean age 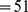 years) shows higher values in the global efficiency and lower values difference in the local efficiency compared with the old group (mean age 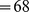 years). These evidences may suggest that age-related alterations in cortical functional networks can be related to structural deficits. Honey 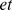 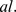 [57] have found that the spontaneous neuronal dynamics can be structured at multiple temporal scales, proposing a tight association between functional and structural networks. Thus, it could be speculated that the age-related alterations in functional networks shown here are likely to be caused by structural impairments.

### Betweenness Centrality and Age-related Alterations

In a complex system, node betweenness represents an crucial metric which can be used to determine the relative importance of a node with a network and identify the pivotal nodes in the network [58]. As indicated above (see Results), 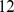 and 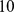 global hub regions (see Table 2, Table 3 and Figure 7) are identified in the older and younger respectively. These hub regions are mainly considered as recently evolved association and primitive limbic regions. It has been proven in the previous study that association regions contribute to the integrity of multiple functional systems, such as memory and attention systems, and are mainly involved in intelligent processing and maintenance of the senior spiritual activity [59]. Meanwhile, limbic regions which are highly interconnected with the prefrontal regions and subcortical regions, are closely related to emotion and a conscious state of mind [59]. Previous studies have reported that identified global hubs were mainly prefrontal and parietal regions, supplying a potential explanation for their well-documented activation by many cognitive functions [7]. In this study, the frontal and parietal regions are also considered as hub regions, especially in older group (see Table 2 and Table 3). Furthermore, although the identified global hubs vary among two age groups, most of these regions are found to show high node betweenness in the functional and structural human brain networks [40], [60], [61], [54], [62]. In addition, it is noted that the substantial discrepancies of identified global hubs between this study and the previous studies can be caused by the different neuroimaging modalities, subjects characteristics and computational methods.

Age-related alterations of hub regions (*e.g.*, SFG and HIP) are also found in this study (see Table 2 and Figure 7). The most of these identified hub regions are association cortices regions (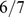 out of 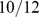) in both age groups. This result is consistent with a previous study that association cortices regions tend to be hubs of the brain functional network regardless of age [17]. From younger group to older group, association cortices show significant changed node betweenness (see Table 2, Table 3 and Table 4). These results support the view that age-related changes are characteristic of association cortex as opposed to primary cortex [63]. We find significant changes in node betweenness with decreasing and increasing in normal aging. This result is also similar to the finding by a previous study which indicated both negative and positive age effects on the regional efficiency in cortical regions [61]. Our finding is also consistent with a previous study that the ageing is associated with significantly reduced nodal efficiency in the frontal neocortex [17]. Above results suggest that frontal which manage movement (see Table 2 and Table 3) plays important roles in contacting information of both groups, but the importance of hippocampus closely related to mental activity is reduced in the older group, indicating the relative degradation of the aged mental activities. The similar findings have been reported in previous studies [63], [64], [65]. Overall, our finding demonstrates age-related changes in the nodal ability to manage information flow of PET networks.

In addition, PET investigations have revealed that the precuneus/posterior cingulate cortex and the medial prefrontal cortex, previously shown to be part of the DMN, display an elevated level of metabolic activity [66]. This result is consistent with our study that some elevated regions in DMN are the hubs (e.g. PCG.R, PCUN.R) which show nodal ability to manage the whole-brain PET network. Furthermore, a former study [20] has reported that magnitude of DMN co-activation in some regions (e.g. HIP.L and SFGmed.R) decreases with normal aging. In this study, these regions also show decreased centrality in older group. Hence, our whole-brain PET networks reveal similar regional characteristics to the previous DMN studies.

### Topological Vulnerability in Functional Networks in Older Group

It has been demonstrated that small-world brain networks with embedded hubs exhibit surprising resilience to random failures and targeted attacks [40], [46]. Assuming that dynamic behavior of a network is strongly related to its fundamental configuration, it seems reasonable to suppose that the changes in network parameters reflect the disruptions in the general performance of the network such as stability and robustness. This hypothesis is supported by our results that the networks in older group are significantly vulnerable to targeted attacks on its pivotal nodes (hub regions) compared with younger group. The reduced topological stability is associated with senescent functional organization in older group such as small-world architecture, and nodal centrality shown previously. Moreover, former studies have reported the vulnerable topological organization of brain structural cortical networks in patients with AD [19]. Thus, this evidence from our study suggests that normal senescence has risk for AD.

### Methodology

In this study, we constructed large-scale human brain functional networks *via* PET data. It is reasonable to conclude that cerebral glucose metabolism from PET data represent the regional functional activity [26], [27]. Effective connectivity between PET regions has also been revealed in previous studies about investigating brain functional systems [30], [31].

According to results about methodological robustness, the similar small-world parameters (see Figure 9) are obtained in responses to the decrease of the sample size. Small-world properties in both groups (Figure 9 **A** and **B**), reduced global efficiency (Figure 9 **D**) and increased local efficiency (Figure 9 **D**) in older group are also found. Thus, it is reasonable to consider that this method demonstrates sufficiently reliable. While this study was a cross-sectional study, a longitudinal analysis would also be useful to investigate the changes of functional brain networks with normal aging. In future studies of functional brain network development, younger individuals are expected to be involved in farther experiments.

## Conclusion

As mentioned above, by using PET data with graph theory analysis, this study demonstrates age-related changes in the topological organization of large-scale functional brain networks constructed *via* a robust method. These results indicate that normal senescence has a notable effect on the topological organization of functional brain networks. Our findings are also compatible with previous studies about the small-world properties, hub regions and network robustness of brain functional and structural networks, thus enhancing our understanding of the underlying physiology of normal aging in human brain.

## Supporting Information

Table S1 **90 regions of interest included in AAL-atlas.**
(PDF)
