# Supplementary material for: Changes in Topological Organization of Functional PET Brain Network with Normal Aging
Source: PLoS One. 2014 Feb 20;9(2):e88690. doi: 10.1371/journal.pone.0088690 (PMC3930631; doi:10.1371/journal.pone.0088690)
Supplement: Table S1 — 90 regions of interest included in AAL-atlas. (PDF) [file pone.0088690.s001.pdf]

Table S1. Regions of interest included in AAL-atlas

Labels	Regions	Regions	abbr.
1	Precentral_L	Precental gyrus	PreCG.L
2	Precentral_R	Precental gyrus	PreCG.R
3	Frontal_Sup_L	Superior frontal gyrus, dorsolateral	SFGdor.L
4	Frontal_Sup_R	Superior frontal gyrus, dorsolateral	SFGdor.R
5	Frontal_Sup_Orb_L	Superior frontal gyrus, orbital part	ORBsup.L
6	Frontal_Sup_Orb_R	Superior frontal gyrus, orbital part	ORBsup.R
7	Frontal_Mid_L	Middle frontal gyrus	MFG.L
8	Frontal_Mid_R	Middle frontal gyrus	MFG.R
9	Frontal_Mid_Orb_L	Middle frontal gyrus, orbital part	ORBmid.L
10	Frontal_Mid_Orb_R	Middle frontal gyrus, orbital part	ORBmid.R
11	Frontal_Inf_Oper_L	Inferior frontal gyrus, opercular part	IFGoperc.L
12	Frontal_Inf_Oper_R	Inferior frontal gyrus, opercular part	IFGoperc.R
13	Frontal_Inf_Tri_L	Inferior frontal gyrus, triangular part	IFGtriang.L
14	Frontal_Inf_Tri_R	Inferior frontal gyrus, triangular part	IFGtriang.R
15	Frontal_Inf_Orb_L	Inferior frontal gyrus, orbital part	ORBinf.L
16	Frontal_Inf_Orb_R	Inferior frontal gyrus, orbital part	ORBinf.R
17	Rolandic_Oper_L	Rolandic operculum	ROL.L
18	Rolandic_Oper_R	Rolandic operculum	ROL.R
19	Supp_Motor_Area_L	Supplementary motor area	SMA.L
20	Supp_Motor_Area_R	Supplementary motor area	SMA.R
21	Olfactory_L	Olfactory cortex	OLF.L
22	Olfactory_R	Olfactory cortex	OLF.R
23	Frontal_Sup_Medial_L	Superior frontal gyrus, medial	SFGmed.L
24	Frontal_Sup_Medial_R	Superior frontal gyrus, medial	SFGmed.R
25	Frontal_Mid_Orb_L	Superior frontal gyrus, medial orbital	ORBsupmed.L
26	Frontal_Mid_Orb_R	Superior frontal gyrus, medial orbital	ORBsupmed.R
27	Rectus_L	Gyrus rectus	REC.L

28	Rectus_R	Gyrus rectus	REC.R
29	Insula_L	Insula	INS.L
30	Insula_R	Insula	INS.R
31	Cingulum_Ant_L	Anterior cingulate and paracingulate gyri	ACG.L
32	Cingulum_Ant_R	Anterior cingulate and paracingulate gyri	ACG.R
33	Cingulum_Mid_L	Median cingulate and paracingulate gyri	DCG.L
34	Cingulum_Mid_R	Median cingulate and paracingulate gyri	DCG.R
35	Cingulum_Post_L	Posterior cingulate gyrus	PCG.L
36	Cingulum_Post_R	Posterior cingulate gyrus	PCG.R
37	Hippocampus_L	Hippocampus	HIP.L
38	Hippocampus_R	Hippocampus	HIP.R
39	ParaHippocampal_L	Parahippocampal gyrus	PHG.L
40	ParaHippocampal_R	Parahippocampal gyrus	PHG.R
41	Amygdala_L	Amygdala	AMYG.L
42	Amygdala_R	Amygdala	AMYG.R
43	Calcarine_L	Calcarine fissure and surrounding cortex	CAL.L
44	Calcarine_R	Calcarine fissure and surrounding cortex	CAL.R
45	Cuneus_L	Cuneus	CUN.L
46	Cuneus_R	Cuneus	CUN.R
47	Lingual_L	Lingual gyrus	LING.L
48	Lingual_R	Lingual gyrus	LING.R
49	Occipital_Sup_L	Superior occipital gyrus	SOG.L
50	Occipital_Sup_R	Superior occipital gyrus	SOG.R
51	Occipital_Mid_L	Middle occipital gyrus	MOG.L
52	Occipital_Mid_R	Middle occipital gyrus	MOG.R
53	Occipital_Inf_L	Inferior occipital gyrus	IOG.L
54	Occipital_Inf_R	Inferior occipital gyrus	IOG.R
55	Fusiform_L	Fusiform gyrus	FFG.L
56	Fusiform_R	Fusiform gyrus	FFG.R
57	Postcentral_L	Postcentral gyrus	PoCG.L
58	Postcentral_R	Postcentral gyrus	PoCG.R
59	Parietal_Sup_L	Superior parietal gyrus	SPG.L
60	Parietal_Sup_R	Superior parietal gyrus	SPG.R
61	Parietal_Inf_L	Inferior parietal, but supramarginal and angular gyri	IPL.L
62	Parietal_Inf_R	Inferior parietal, but supramarginal and angular gyri	IPL.R

63	SupraMarginal_L	Supramarginal gyrus	SMG.L
64	SupraMarginal_R	Supramarginal gyrus	SMG.R
65	Angular_L	Angular gyrus	ANG.L
66	Angular_R	Angular gyrus	ANG.R
67	Precuneus_L	Precuneus	PCUN.L
68	Precuneus_R	Precuneus	PCUN.R
69	Paracentral_Lobule_L	Paracentral lobule	PCL.L
70	Paracentral_Lobule_R	Paracentral lobule	PCL.R
71	Caudate_L	Caudate nucleus	CAU.L
72	Caudate_R	Caudate nucleus	CAU.R
73	Putamen_L	Lenticular nucleus, putamen	PUT.L
74	Putamen_R	Lenticular nucleus, putamen	PUT.R
75	Pallidum_L	Lenticular nucleus, pallidum	PAL.L
76	Pallidum_R	Lenticular nucleus, pallidum	PAL.R
77	Thalamus_L	Thalamus	THA.L
78	Thalamus_R	Thalamus	THA.R
79	Heschl_L	Heschl gyrus	HES.L
80	Heschl_R	Heschl gyrus	HES.R
81	Temporal_Sup_L	Superior temporal gyrus	STG.L
82	Temporal_Sup_R	Superior temporal gyrus	STG.R
83	Temporal_Pole_Sup_L	Temporal pole: superior temporal gyrus	TPOsup.L
84	Temporal_Pole_Sup_R	Temporal pole: superior temporal gyrus	TPOsup.R
85	Temporal_Mid_L	Middle temporal gyrus	MTG.L
86	Temporal_Mid_R	Middle temporal gyrus	MTG.R
87	Temporal_Pole_Mid_L	Temporal pole: middle temporal gyrus	TPOmid.L
88	Temporal_Pole_Mid_R	Temporal pole: middle temporal gyrus	TPOmid.R
89	Temporal_Inf_L	Inferior temporal gyrus	ITG.L
90	Temporal_Inf_R	Inferior temporal gyrus	ITG.R
